# Effects of 28 days of two creatine nitrate based dietary supplements on bench press power in recreationally active males

**DOI:** 10.1186/1550-2783-12-S1-P17

**Published:** 2015-09-21

**Authors:** E Galvan, A O'Connor, Y C Goodenough, R Dalton, K Levers, N Barringer, M Cho, P Jung, M Greenwoord, C Rasmussen, PS Murano, C P Earnest, R Kreider

**Affiliations:** 1 Exercise & Sport Nutrition Lab, Texas A&M University, College Station, TX, USA; 2 Institute for Obesity and Program Evaluation, Texas A&M University, College Station, TX, USA; 3 Research & Development, Nutrabolt Corp., Bryan, TX, USA

## Background

Athletes use ergogenic aids in an attempt to increase training-adaptations, which serves to enhance their performance during competition. Creatine monohydrate is one of the most studied ergogenic aids. Although many studies have reported the efficacy and effectiveness of creatine monohydrate supplement manufacturers continually introduce newer forms of creatine into the marketplace. The newer forms of creatine purport to be more effective than creatine monohydrate alone. However, there is little evidence to support most manufacturers' claims.

## Methods

We examined 28d of randomly assigned (1) placebo (PL), (2) Creatine monohydrate (CrM; 3 g), (3) creatine nitrate (CrN; 1 g CrM; 0.5 g N) and (4) CrN2X (2 g CrM; 1.0 g N) on bench press performance. Participants (N = 48; 21 ± 3 yrs) presented for fasting (12 h) testing after abstaining from exercise and alcohol for 48 h. Performance (reps at 70% of bench press 1 RM) was measured using a Tendo Fitrodyne at 0 & 28d and analyzed by MANOVA or one-way ANOVA. Mean changes (95% CI) were reported.

## Results

We previously reported (*FASEB J, 29(1):LB248, 2015*) that all treatment groups increased bench press repetitions after 28d of supplementation; however, total work (reps × weight lifted) during bench press was greater at 28d for CrN2X (294.6 lbs; 95% CI, 196, 393) vs. CrN (164.2 lbs; 95% CI, 25, 304) and PL (187.1 lbs; 95% CI, 37, 336, both p = 0.02). MANOVA univariate analysis of power data indicated a significant time effect with all power output variables (i.e., peak power (PP), average power (AP), and average velocity (AV)). No significant group by time effects were observed among groups. One-way ANOVA of the 3^rd ^set of exercise performed to exhaustion revealed no significant differences among groups in changes from baseline after 28d of supplementation. However, pairwise comparison of 95% CIs revealed a significant difference in peak power and average power between CrN2X (522.8 W; 95% CI, 473.5, 572.2) and PL (422.9 W; 95% CI, 386.6, 499.1, p = 0.037) and CrN2X (470.3 W; 95% CI 422.1, 518.5) and PL (386.1 W; 95% CI, 331.1, 441.0, p = 0.025), respectively. Average power was also significantly different between CrN2X (470.3 W; 95% CI 422.1, 518.5) and CrN (384.0 W; 95% CI, 335.8, 432.2, p = 0.014). Average velocity during bench press test was also significantly different between CrN (0.629 m/s; 95% CI, 0.572, 0.686) and PL (0.525 m/s; 95%, 0.460, 0.590, p = 0.02).

## Conclusion

Results suggest some ergogenic value of consuming these types of creatine containing pre-workout supplements on bench press power adaptations during training in comparison to PL responses.

